# Kupffer Cells Undergo Fundamental Changes during the Development of Experimental NASH and Are Critical in Initiating Liver Damage and Inflammation

**DOI:** 10.1371/journal.pone.0159524

**Published:** 2016-07-25

**Authors:** D. T. Reid, J. L. Reyes, B. A. McDonald, T. Vo, R. A. Reimer, B. Eksteen

**Affiliations:** 1 Snyder Institute for Chronic Diseases, Cumming School of Medicine, University of Calgary, Calgary, Alberta, Canada; 2 Laboratorio de Inmunología Experimental y Regulación de la Inflamación Hepato-intestinal, UBIMED, FES Iztacala, UNAM, Mexico; 3 Department of Ecosystem and Public Health, Faculty of Veterinary Medicine, University of Calgary, Calgary, Alberta, Canada; 4 Department of Biochemistry & Molecular Biology, Cumming School of Medicine, University of Calgary, Calgary, Alberta, Canada; 5 Faculty of Kinesiology, University of Calgary, Calgary, Alberta, Canada; RWTH Aachen, GERMANY

## Abstract

Non-alcoholic fatty liver disease has become the leading liver disease in North America and is associated with the progressive inflammatory liver disease non-alcoholic steatohepatitis (NASH). Considerable effort has been made to understand the role of resident and recruited macrophage populations in NASH however numerous questions remain. Our goal was to characterize the dynamic changes in liver macrophages during the initiation of NASH in a murine model. Using the methionine-choline deficient diet we found that liver-resident macrophages, Kupffer cells were lost early in disease onset followed by a robust infiltration of Ly-6C^+^ monocyte-derived macrophages that retained a dynamic phenotype. Genetic profiling revealed distinct patterns of inflammatory gene expression between macrophage subsets. Only early depletion of liver macrophages using liposomal clodronate prevented the development of NASH in mice suggesting that Kupffer cells are critical for the orchestration of inflammation during experimental NASH. Increased understanding of these dynamics may allow us to target potentially harmful populations whilst promoting anti-inflammatory or restorative populations to ultimately guide the development of effective treatment strategies.

## Introduction

Non-alcoholic fatty liver disease (NAFLD) has emerged as the leading liver disease in North America[1]. Considered the hepatic manifestation of obesity, NAFLD includes a spectrum of liver diseases including the progressive inflammatory disease non-alcoholic steatohepatitis (NASH). Numerous studies have been carried out to understand the role of both resident and recruited immune cells contributing to the progression of NASH. During the development of steatohepatitis, recruited monocytes and neutrophils as well as T cells and iNKT cells have been found to be contributors to the progression of NASH[2, 3]. Tissue-resident macrophages known as Kupffer cells (KC) have been associated with the production of proinflammatory mediators such as TNF-α and IL-1β as well as anti-inflammatory mediators including IL-10 and arginase[4, 5]. Controversy over the identification and function of tissue-resident and recruited macrophage populations continues to hamper our understanding of the critical role of these cells in NASH.

Considerable discussion remains centered around the different macrophage populations present in the liver in health and disease. At steady state, conventional Kupffer cells are the dominant tissue-resident macrophage and reside in the liver sinusoids contributing to pathogen clearance and tissue homeostasis[6]. They are often characterized by high F4/80 surface expression and are negative for chemokine receptors including CX3CR1 and CCR2[7]. A smaller population of monocyte-derived macrophages expressing CX3CR1 have also been identified in livers of healthy mice[7]. However, APAP-induced acute liver injury and viral infection can result in the loss of the resident population of KC [7, 8]. In turn, Kupffer cells are replaced by a robust infiltration of circulating monocytes that can be defined as Ly-6C^hi^CD11b^hi^MHC II^neg^CX3CR1^+^[7]. Recruitment of CCR2^+^ monocytes from the bone marrow may promote proinflammatory monocyte accumulation in the liver that has been shown to lead to the development of fibrosis[9]. On the other hand CCR2 deficiency has been linked to a reduction in monocyte recruitment and impaired tissue repair in the liver[7]. Use of the CCR2/CCR5 antagonist, Cenicriviroc (CVC) has been shown to have potent anti-inflammatory effects in mice and is currently being evaluated in a phase 2b clinical trial in patients with NASH and fibrosis[10]. In a murine model of alcoholic liver disease, polarization of Kupffer cells towards an anti-inflammatory phenotype resulted in the release of arginase and apoptosis of iNOS-expressing proinflammatory KC[5]. Development of liver fibrosis has been associated with a Ly-6C^hi^ recruited monocyte population whereas tissue restoration is associated with Ly-6C^lo^ population[11]. Microarray analysis of these Ly-6C^+^ populations revealed numerous differences in gene expression for matrix metalloproteinases, growth factors and phagocytosis factors[11]. Continued characterization of resident and recruited macrophage populations is required to understand the underlying pathogenesis of NASH.

Although numerous studies in patients with NASH have been conducted, these studies remain a challenge due to the need for diagnostic liver biopsy that is costly and invasive [12]. Therefore the use of animal models is key to help identify the underlying disease etiology[13]. To study hepatic injury and inflammation, the methionine and choline deficient (MCD) diet is one of the most commonly used mouse model of NASH [14]. Although MCD diet has been used to evaluate disease mechanisms and potential treatment strategies little is known about the initiation of inflammation in the model. The contribution of liver-resident macrophages to the initiation and progression of NASH remains unclear.

Given the current gaps in our understanding of NASH progression, our objective was to characterize the dynamic changes in tissue-resident macrophages and recruited immune cells in a longitudinal manner during MCD diet-induced murine NASH.

## Methods

### Animals and Housing

The study protocol was approved by The University of Calgary Animal Care Committee and conformed to the *Guide for the Care and Use of Laboratory Animals*. All studies were carried out using male, C57/bl6 mice (approximately 8 weeks of age) raised in the mouse colony at the University of Calgary (Health Sciences Animal Research Centre, Calgary, AB, Canada). Animals were kept on a 12-hour light/dark cycle in a temperature and humidity control room with free access to food and drinking water.

### Murine model of non-alcoholic steatohepatitis

Feeding mice a diet deficient in methionine and choline (MCD diet) is the most commonly used mouse model to study the inflammation associated with NASH (4.2kcal/g; MP Biomedicals, Solon, OH, USA) [15]. Mice fed MCD diet consistently develop steatosis, inflammation and hepatocellular injury in a short period of time (21 days) making it the standard dietary protocol to study murine NASH. Histological comparison of a patient biopsy and a liver section from a mouse fed MCD diet for three weeks demonstrates similar disease traits including steatosis, ballooning hepatocytes and inflammatory cell foci[16]. Ballooning hepatocytes refers to a morphological change in lipid-laden hepatocytes that is indicative of damaged and apoptotic hepatocytes and in combination with steatosis and inflammation is correlated with worse liver disease [17]. Mice may develop liver fibrosis if MCD diet is continued beyond three weeks (S1 Fig). Since MCD diet feeding can result in weight loss body weight was recorded weekly. If mice lost greater than 30% of initial body weight they were removed from the study. No mice lost greater than 20% of initial body weight in the current study. Given that the obese phenotype is not present in the MCD diet model, we also examined a high fat-high sucrose (HF/HS) feeding model where mice were fed chow or HF/HS diet for 13 weeks (4.6kcal/g; Dyets Inc, Bethlehem, PA, USA). It was anticipated that this common dietary potentiator of metabolic disease would allow us to assess early changes in tissue-resident macrophages.

### Macrophage depletion with liposomal clodronate

Liposome-encapsulated clodronate was administered IV through the tail vein at a concentration of 0.026mol/L to deplete macrophages[18] (n = 8/group; Haarlem, Netherlands). PBS was used as the vehicle control[19]. Macrophage depletion was confirmed by flow cytometry analysis of reduction in F4/80+ cells.

### Hepatocellular injury

Degree of hepatocellular damage was indirectly quantified by measuring circulating alanine aminotransferase (ALT) levels from serum collected via cardiac puncture (Calgary Laboratory Services, Calgary, AB, Canada). Following blood collection mice were euthanized by cervical dislocation while under deep anesthetic (Isoflurane, Fresenius Kabi, Richmond Hill, ON, Canada).

### NAFLD Activity Score (NAS) for disease severity

The NAS is a valid and reliable histological scoring tool that was developed for the purpose of numerically rating the progression of NAFLD in patient liver biopsies [20]. The scoring includes measurement of steatosis grade (0-no steatosis, 1 - <50% steatosis, 2 - > 50% steatosis, 3 - >50% steatosis + microvesicular steatosis), hepatocyte ballooning (0- none, 1- <66%, 2- >66% hepatocyte involvement) and inflammation (0-no foci, 1- <2 foci, 2–2–4 foci, 3- +4 foci). A score of 4 or above signifies the development of NASH [21].

### RNA extraction and real-time quantitative PCR analysis

RNA was extracted from snap frozen tissue using RNeasy Mini Kit (Qiagen, Toronto, ON, Canada). The concentration of total RNA was quantified by NanoDrop 2000 (Thermo Fisher Scientific Inc, Mississauga, ON, Canada) followed by reverse transcription using the RT^2^ first strand cDNA synthesis kit for RT-PCR (Qiagen, Toronto, ON, Canada). The resultant cDNA was amplified using primers generated through the Universal ProbeLibrary (Roche Diagnostics, Laval, QC, Canada) and synthesized by the University of Calgary Core DNA Services (Calgary, AB, Canada). A StepOne Real-Time PCR System was used for the real-time PCR reactions (Thermo Fisher Scientific Inc, Mississauga, ON, Canada) and FAM detection probes (Roche Diagnostics, Laval, QC, Canada). Data was normalized to β-actin for genes of interest in the liver. The 2^-ΔCT^ method [ΔC_T_ = C_T_ (gene of interest)–C_T_ (reference gene)] was utilized for the data analysis where threshold cycle (C_T_) indicates the fractional cycle number at which the amount of amplified target reaches a fixed threshold [22]. The following primer sequences were used: α-smooth muscle actin (SMA) 5’-CTCTCTTCCAGCCATCTTTCAT-3’, 5’-CGTAGGTGCTTTGGTGGATAT-3’β-actin 5’-CTAAGGCCAACCGTGAAA-3’, 5’-AGGGACATACGGAGACCA-3’

### Leukocyte recruitment

Flow cytometry was employed for the characterization of resident and recruited leukocytes to the liver during health and disease. Briefly, fresh liver tissue was placed in cold phosphate buffered saline containing 0.5% fetal calf serum and 2mM EDTA and immediately homogenized using a gentleMACS tissue dissociator (Miltenyi Biotec Inc, Auburn CA, USA). Tissue homogenate was filtered through a 60 micron mesh to achieve a single cell suspension. Following centrifugation, cells were layered on a 33%/77%Percoll gradient and spun at room temperature at 600rcf for 20 minutes (GE Healthcare Bio-Sciences AB, Uppsala, Sweden). Leukocytes were removed from the gradient, pelleted and resuspended in cold FACS buffer (PBS, 0.5% BSA and 2mM EDTA) followed by incubation with antibodies for flow cytometry at 4°C for 2 hours. The antibodies used include Ly-6C, CD11b, CD3, NK1.1 (BD Biosciences, Mississauga, ON, Canada), F4/80 (eBioscience, San Diego, CA, USA), CD4, CD8 (Abcam, Toronto, ON, Canada), CCR2 and CX3CR1 (R&D Systems, Minneapolis, MN, USA). Data was collected on a FACS Aria II (BD Biosciences, Mississauga, ON, Canada) using facs diva software and analyzed by Kaluza software (Beckman Coulter, Mississauga, ON, Canada).

### Multiplex gene expression

Gene expression analysis was carried out on liver macrophages using fluorescent activated cell sorting (FACS) followed by mRNA extraction (BD Aria Fusion). Briefly, cells were sorted into cold PBS, pelleted and resuspended in TRIzol for cell lysis followed by chloroform extraction and ethanol precipitation (Thermo Fisher Scientific, Mississauga, ON, Canada). Isolated mRNA was quantified using Nanodrop (Thermo Fisher Scientific, Mississauga, ON, Canada). Normalized concentrations of mRNA were then hybridized and sequenced using the preassembled Mouse Inflammation v2 gene profiling CodeSet and nCounter platform according to the manufacturers protocol (Nanostring Technologies, Seattle, WA, USA). Data was analyzed using nSolver software provided by Nanostring Technologies.

### KEGG network analysis

Changes in gene expression from the sequencing data were mapped into biological pathways to create a network analysis of known cellular functions using the Kyoto Encyclopedia for Genes and Genomes (KEGG) pathway databases [23, 24]. A mapping file was created to colour code these genes through the KEGG Pathway mapper.

### Statistical Analyses

All data are expressed as mean ± SEM. Statistical analysis of data was carried out using student’s T test or one-way analysis of variance (ANOVA) followed by Tukey’s post-hoc test as indicated using GraphPad Prism v.6. Differences were considered statistically significant at P<0.05.

## Results

### Liver injury precedes steatohepatitis in mice fed MCD diet

**Fig 1:**
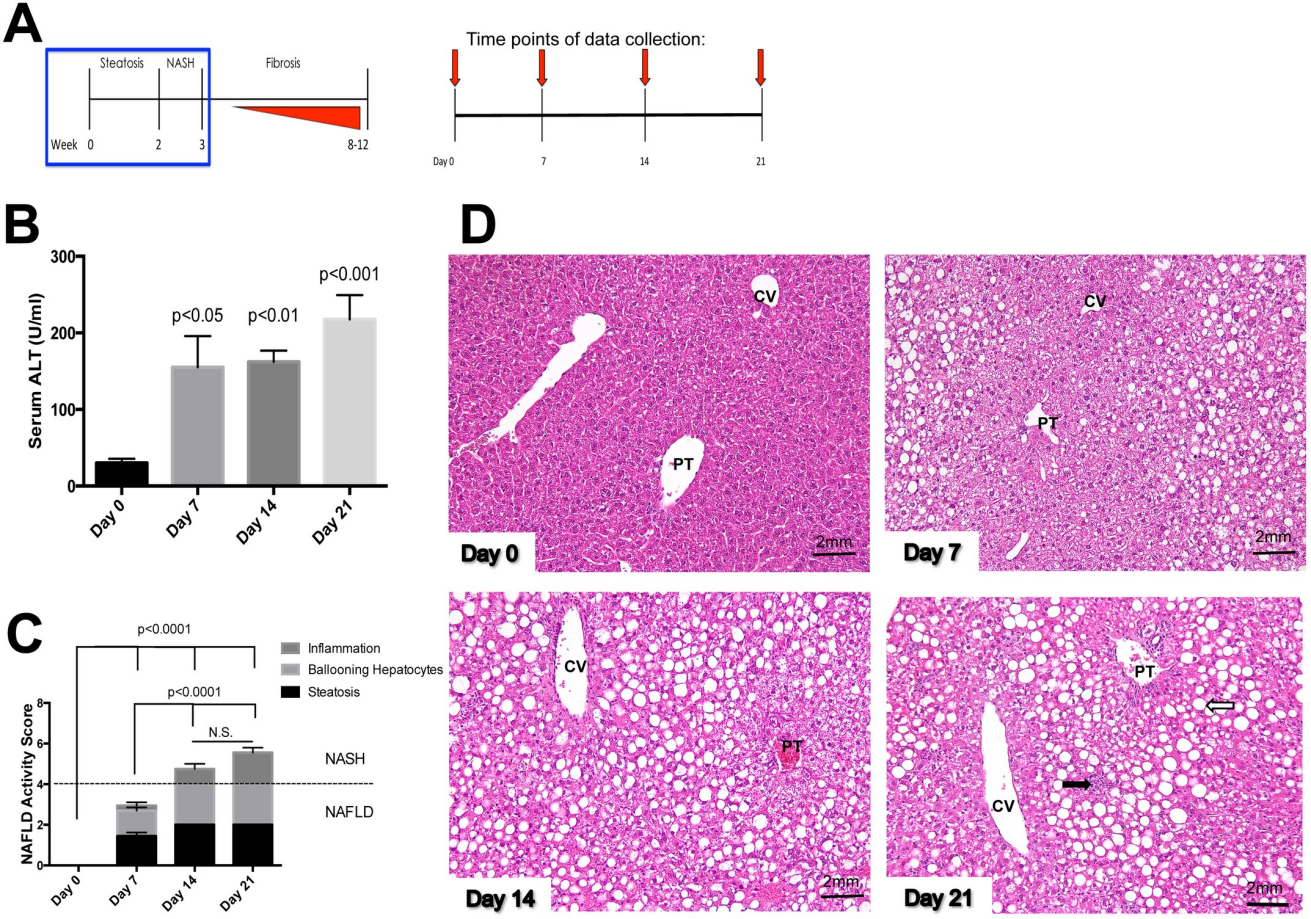
**MCD diet feeding results in liver injury and inflammation** (A) Timeline of the development of steatosis, NASH and fibrosis in mice fed MCD diet. Schematic represents timeline of data collection (red arrows) during MCD diet feeding treatment (n = 7-9/group). (B) ALT levels are significantly elevated after 7 (p<0.05), 14 (p<0.01) and 21 days (p<0.001) of feeding compared to D0. (C) Mice fed MCD diet for 7, 14 and 21 days had a significantly greater NAS score compared to mice at D0 (p<0.0001). Mice fed MCD diet for 14 and 21 days developed steatohepatitis compared to mice fed MCD diet for 7 days (p<0.0001). (D) Representative images of H&E stained liver sections following 0, 7, 14 or 21 days of MCD diet feeding. White arrow indicates lipid accumulation and black arrow indicates inflammatory foci. 20x magnification, PT: portal tract, CV: central vein.

We examined the development of steatosis and the progression to NASH by assessing liver injury and inflammation after 7, 14 and 21 days of MCD diet feeding (Fig 1A). Serum ALT levels were elevated after 7 days of MCD diet feeding compared to mice fed standard chow diet on Day 0 (Fig 1B, p<0.05) and continued to rise after 14 and 21 days of MCD diet feeding (Fig 1B, p<0.01 and p<0.001, respectively). Next we scored liver tissue sections using the NAFLD activity score (NAS) to evaluate the histopathology of NASH. After 7 days of MCD diet feeding, mice developed steatosis with evidence of ballooning hepatocytes compared to chow fed mice (Fig 1C, NAS = 3, p<0.0001). Mice fed MCD diet for 14 days develop steatohepatitis with an NAS score >4 that increased to >5 after 21 days of MCD diet feeding (Fig 1C, p<0.0001 compared to chow fed mice). Representative histology images of liver sections show progressive macrovesicular lipid accumulation within hepatocytes as early as day 7 located predominately in the portal region after 21 days of MCD diet feeding (Fig 1D). Evidence of hepatocellular ballooning and liver injury as well as the infiltration of inflammatory cells indicates the development of necroinflammation at day 21 (Fig 1D).

### MCD diet feeding results in a loss of conventional Kupffer cells and emergence of monocyte-derived macrophages

**Fig 2:**
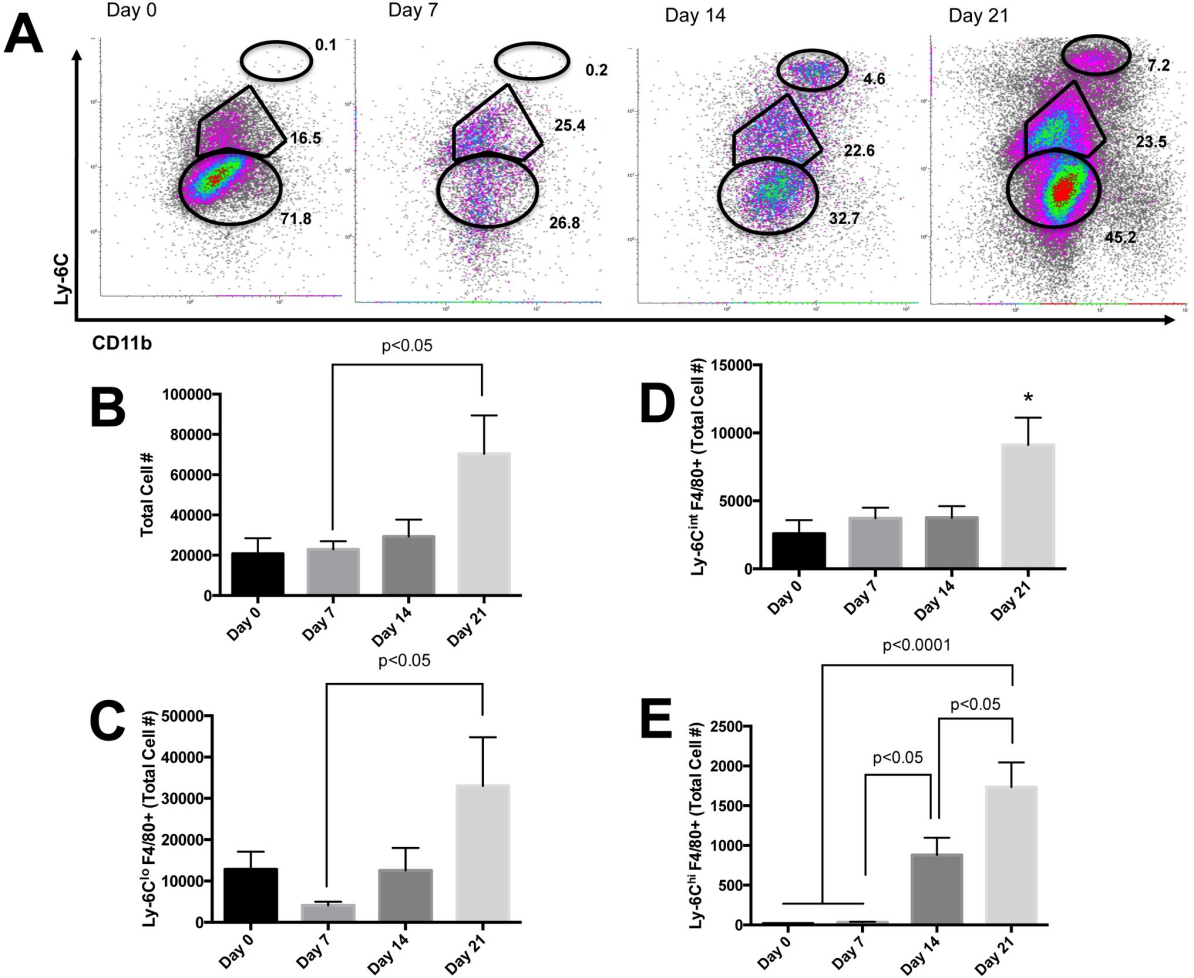
Changes in resident and recruited macrophage populations. (A) Effect of MCD diet feeding over 21 days on resident macrophages and recruited populations of monocyte-derived macrophages (gated on F4/80+). (B) MCD diet feeding resulted in significantly more cellular infiltration after 21 days compared to D7 (p<0.05). (C) One week of MCD diet feeding resulted in significantly reduced numbers of Ly-6C^lo^ liver-resident macrophages compared to D21 (p<0.05). (D) Numbers of Ly-6C^int^ monocyte-derived macrophages was significantly elevated after 21 days of MCD diet feeding (p<0.05). (E) Recruitment of Ly-6C^hi^ monocyte-derived macrophages was significantly elevated between D7 and D14 (p<0.05) and after 21 days of MCD diet feeding compared to D0 and D7 (p<0.0001) and D14 (p<0.05).

To further understand how MCD diet feeding affects resident cells and the recruitment of inflammatory cells leading to the development of necroinflammation, we assessed changes in both resident and recruited macrophage populations. Prior to MCD diet feeding, liver-resident macrophages, Kupffer cells, can be identified as Ly-6C^lo^ CD11b^lo^ F4/80+ (Fig 2A). Following MCD diet treatment, the total number of cells was significantly increased on D21 compared to D7 (Fig 2B, p<0.05). We found a reduction in the number of Kupffer cells at D7 of MCD diet feeding that were replaced by a Ly-6C^lo^ macrophage population on D21 (Fig 2C, p<0.05). Although the percentage of Ly-6C^int^ F4/80+ macrophages did not vary considerably over the course of MCD diet feeding there was a significant increase in the total number of cells after 21 days of MCD diet feeding (Fig 2D, p<0.05). Continued MCD diet feeding resulted in the recruitment of Ly-6C^hi^ F4/80+ cells after D14 compared to D7 (p<0.05) and significantly elevated after 21 days of MCD diet feeding compared to D0 (p<0.0001), D7 (p<0.0001) and D14 (p<0.05) (Fig 2E). There was no difference in liver injury or the number of Kupffer cells in the liver after mice were fed HF/HS diet compared to standard chow (S2 Fig). Therefore, these animals were not studied further.

### Transcriptome analysis reveals differences in resident and recruited liver macrophages during experimental NASH

**Fig 3:**
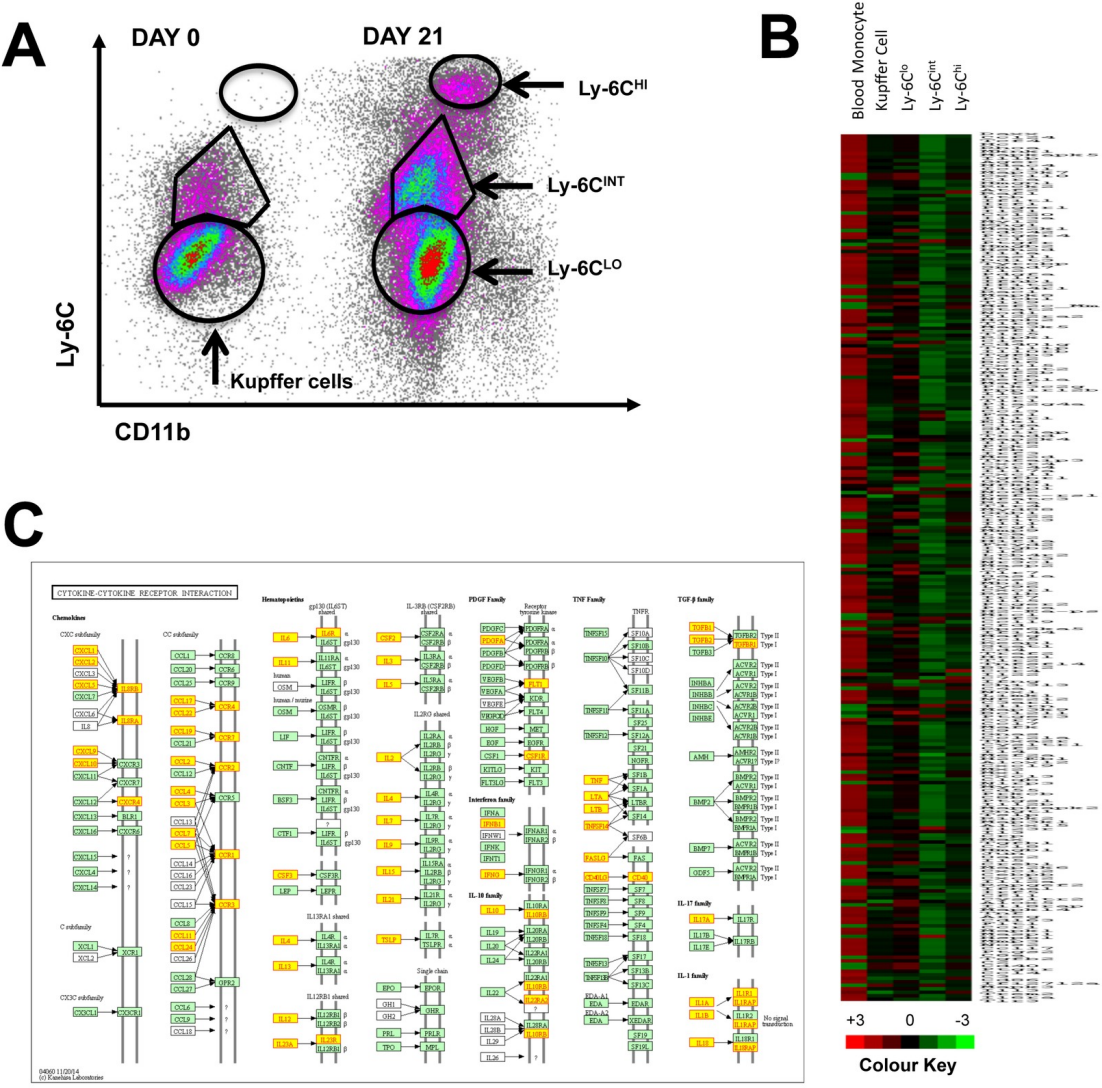
**Transcriptomics analysis of resident and recruited macrophages in the liver during MCD diet treatment** (A) Fluorescence-activated cell sorting gating strategy for liver macrophages. Sorting purity exceeded 90% for all populations. (B) Gene expression profile of inflammation genes associated with steatohepatitis reveals differences between circulating monocytes, resident KC and recruited Ly-6C^+^ macrophage populations. (C) Genes highlighted by yellow boxes demonstrate changes in expression levels, either increased or decreased, in the known cytokine-cytokine receptor interaction pathway between sorted cell populations depicted using the Kyoto Encyclopedia of Genes and Genomes pathway mapper mmu04060.

To explore differences in the gene regulation of both KCs and recruited monocyte-derived macrophages, cell populations were sorted and analyzed using direct multiplexed measurement of gene expression pathways involved in the control of innate and adaptive immunity. Kupffer cells were harvested from healthy mice whereas Ly-6C+ recruited macrophages were FACS-sorted from mice fed MCD diet for 21 days (Fig 3A). Circulating blood monocytes were sorted and analyzed as an additional control group (Fig 3A).

Gene expression analysis showed distinct differences in the cell populations with marked changes in the expression of cytokines, chemokines and the JAK/STAT signaling pathways between blood monocytes, KCs and Ly-6C^+^ macrophages (Fig 3B and Fig 3C). Differences detected include cytokines from the IL-1, IL-2, IL-10, IL-17, TGF-β, TNF, interferon and chemokine families (Fig 3C).

### Recruited monocyte-derived macrophages can be identified by cell-surface expression of CCR2 and CX3CR1

**Fig 4:**
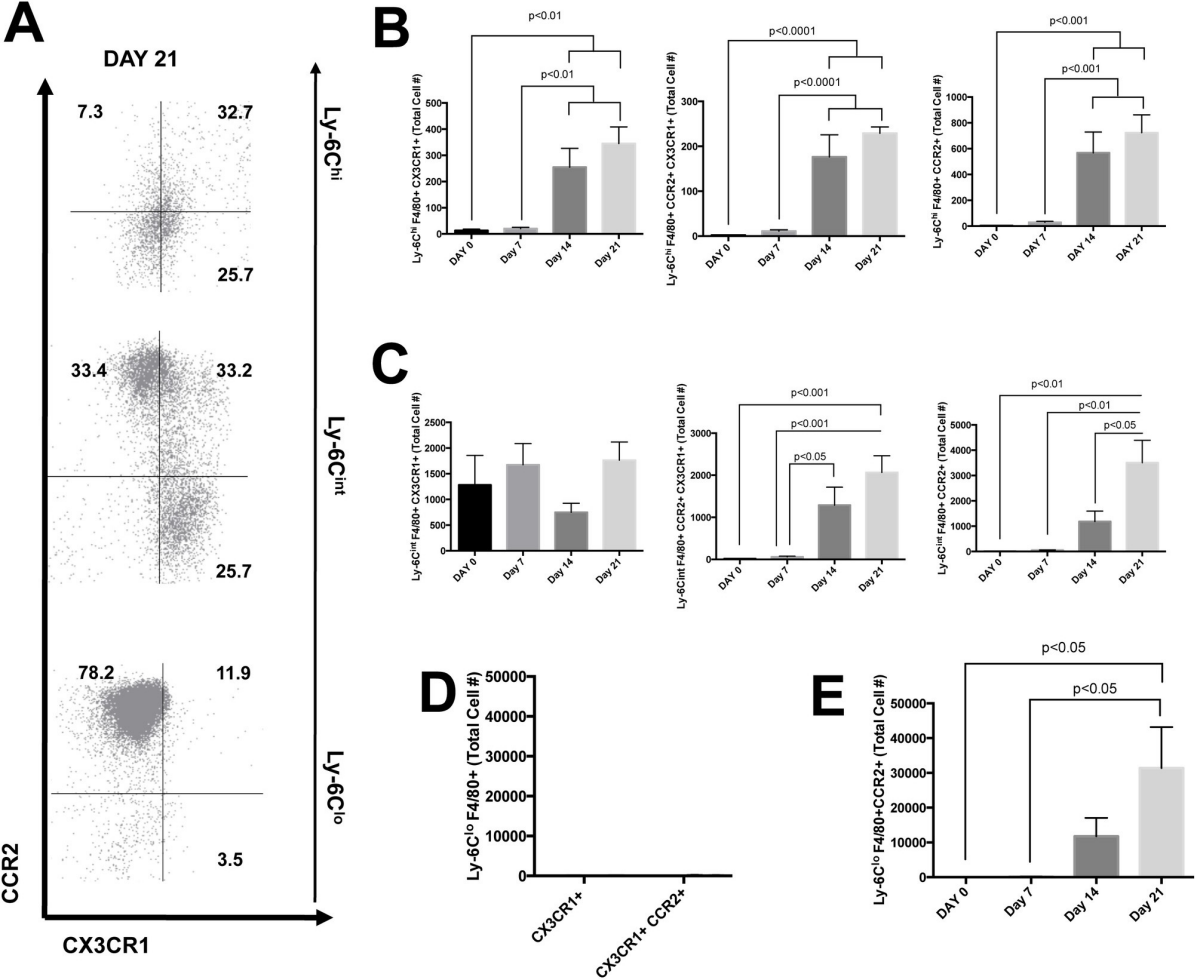
**Cell surface expression of chemokines during steatohepatitis** (A) Recruitment of Ly-6C+ monocyte-derived macrophages can be characterized by CCR2 and CX3CR1 expression. (B) Infiltration of Ly-6C^hi^ monocyte-derived macrophages is partially dependent on CCR2 and CX3CR1. (C) Ly-6C^int^ monocyte-derived macrophages are recruited in part to the liver via CX3CR1 and CCR2. (D) Kupffer cells do not express CCR2 or CX3CR1. (E) Ly-6C^lo^ macrophages are predominately CCR2+ appearing only after 14 days of MCD diet feeding.

The recruitment of circulating immune cells into the liver is an active process mediated in part through chemokine-chemokine receptor interactions[6]. To understand the infiltration of cells during MCD diet feeding we looked at the expression of two chemokine receptors, CCR2 and CX3CR1 that have been linked to driving a proinflammatory immune response as well as the regulation of inflammation and contribution to tissue repair[25] (Fig 4A). As the mice developed steatohepatitis, recruitment of Ly-6C^hi^ macrophages was dependent on both CCR2 and CX3CR1 (Fig 4B). In comparison, there was no difference in the recruitment of Ly-6C^int^ CX3CR1^+^ macrophages during MCD diet feeding whereas Ly-6C^int^ CX3CR1^+^ CCR2^+^ cells were significantly increased following 21 days of MCD diet feeding compared to D0 and D7 (Fig 4C, p<0.001). Similarly, Ly-6C^int^ CCR2^+^ cells were significantly increased after 21 days of MCD diet compared to D0, D7 and D14 (Fig 4C, p<0.01, p<0.01, p<0.05, respectively). Kupffer cells in healthy mice do not express CCR2 or CX3CR1 as expected (Fig 4D). At the peak of liver injury on D21, Ly-6C^lo^ F4/80+ macrophages had abundant expression of CCR2 compared to D0 and D7 of MCD diet feeding (Fig 4E, p<0.05).

### Lymphocytes infiltrate liver tissue after 14 days of MCD diet feeding

**Fig 5:**
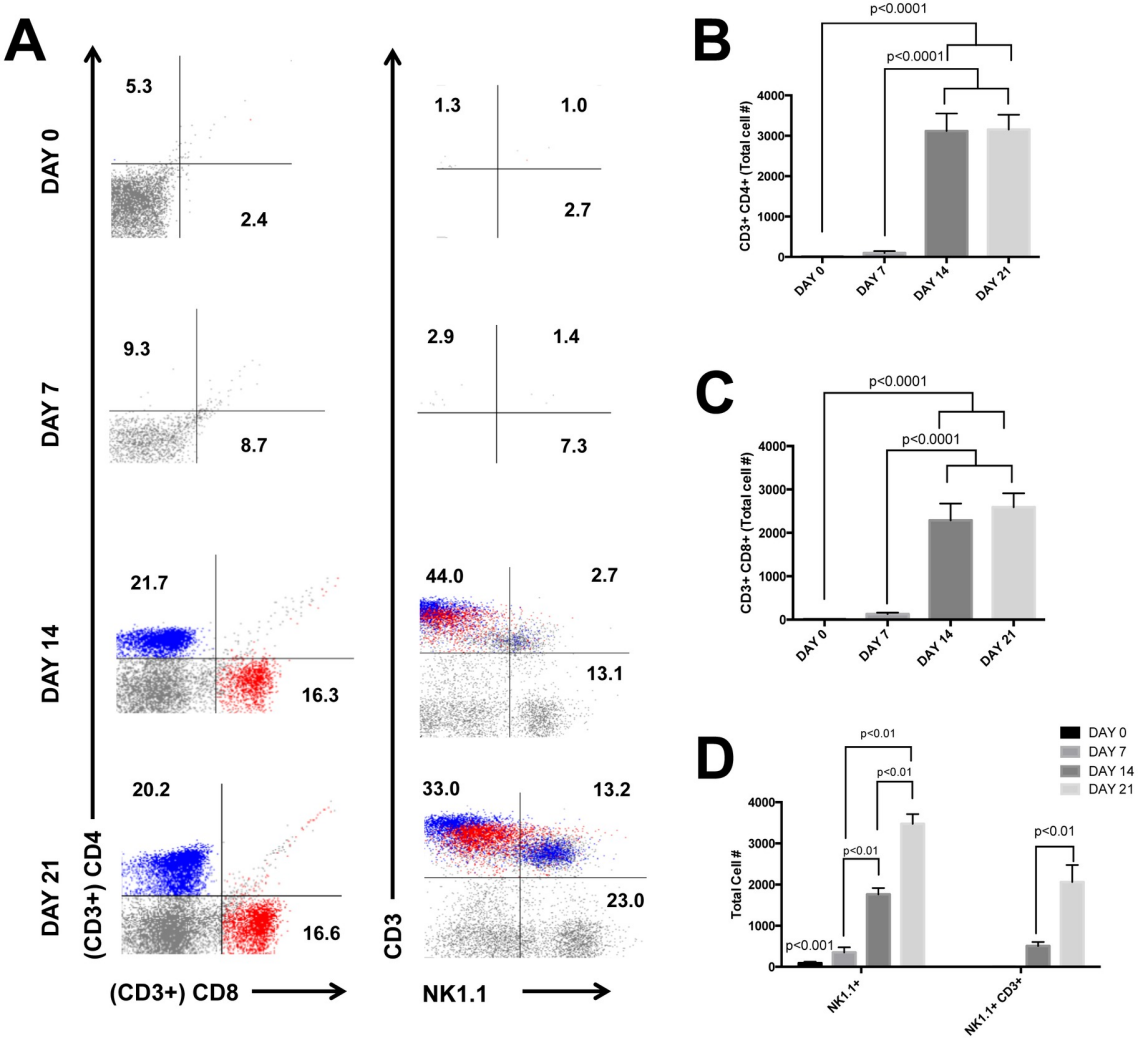
**Leukocyte recruitment is a later event** (A) Emergence of leukocytes is a late event following MCD diet treatment. (B-C) CD4+ and CD8+ T lymphocytes are observed after 14 days of MCD diet feeding. (D) NK cells and NKT cells are more prominent on D21 compared to D14.

Contributing to the mixed leukocyte infiltration during steatohepatitis, lymphocytes can be detected at the peak of liver disease (Fig 5A). We evaluated the pattern of lymphocyte recruitment during MCD diet feeding and found the appearance of CD4+ T cells was a late event beginning after 14 days of MCD diet feeding compared to D0 and D7 (Fig 5B, p<0.0001). Similarly the recruitment of CD8+ T cells occurred after D14 of MCD diet feeding compared to D0 and D7 (Fig 5C, p<0.0001). NK cell numbers were significantly higher at the end of the treatment period compared to D0, D7 and D14 (Fig 5D, p<0.001, p<0.01 and p<0.01, respectively). The number of NKT cells infiltrating the liver are significantly higher on D21 than D14 (Fig 5D, p<0.01).

### Kupffer cell depletion at the initiation of MCD diet feeding protects against liver injury and inflammation

**Fig 6:**
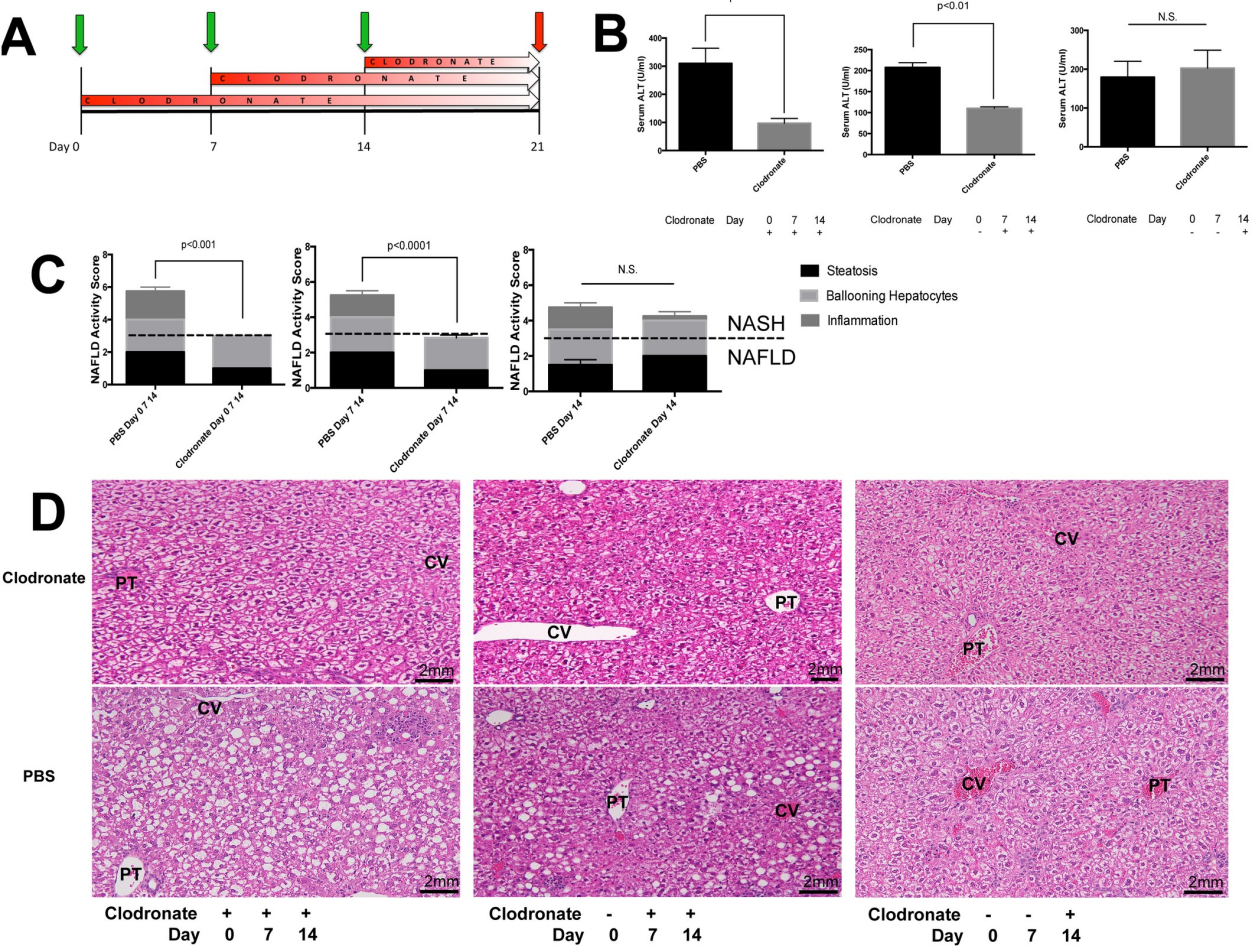
**CLL treatment protects mice from steatohepatitis** (A) Regime of clodronate-loaded liposome (CLL) treatment or PBS initiated at D0, D7 or D14 (green arrows) and repeated on a weekly basis until termination of the study (red arrow). All groups of mice (n = 8/group) were fed MCD diet for the same three-week duration. (B) A significant reduction in serum ALT was observed when CLL was initiated at D0 (p<0.05) and D7 (p<0.01) compared to PBS control whereas no difference was detected when CLL was started at D14. (C) Mice are protected from developing steatohepatitis when CLL is initiated on D0 (p<0.001) and D7 (p<0.0001) compared to PBS control but not when CLL begins at D14 of MCD diet feeding. (D) Representative images of H&E stained liver sections after 21 days of MCD diet feeding following CLL treatment at D0, D7 or D14. 20x magnification, PT: portal tract, CV: central vein.

We have established that the progression of necroinflammation during MCD diet feeding is a dynamic process with both the loss of tissue-resident macrophages and the recruitment of numerous immune cell populations influencing inflammation, tissue damage as well as tissue repair. To further understand the contribution of tissue-resident macrophages driving the recruitment of immune cells leading to liver injury and inflammation we used liposomal-encapsulated clodronate to deplete macrophages within the liver. The clodronate was administered starting at D0, D7 or D14 of MCD diet feeding and continued on a weekly basis until termination of the study at D21 (Fig 6A). Mice were protected from liver injury when macrophages were depleted over the entire course of MCD diet feeding (Fig 6B, p<0.05). There was a significant decrease in serum ALT levels when mice received clodronate starting on D7 of MCD diet feeding (Fig 6B, p<0.01). No difference in serum ALT levels was detected when clodronate was started after 14 days of MCD diet feeding (Fig 6B). Similarly the NAS score was significantly reduced with clodronate treatment beginning at D0 (p<0.01) and starting at D7 (p<0.001) with no difference with treatment starting at D14 (Fig 6C). Mice treated with clodronate over the duration of MCD diet feeding had reduced steatosis and a marked reduction in inflammation compared to control mice (Fig 6D).

### Tissue-resident macrophage depletion reduces Ly-6C+ monocyte-derived macrophage infiltration

**Fig 7:**
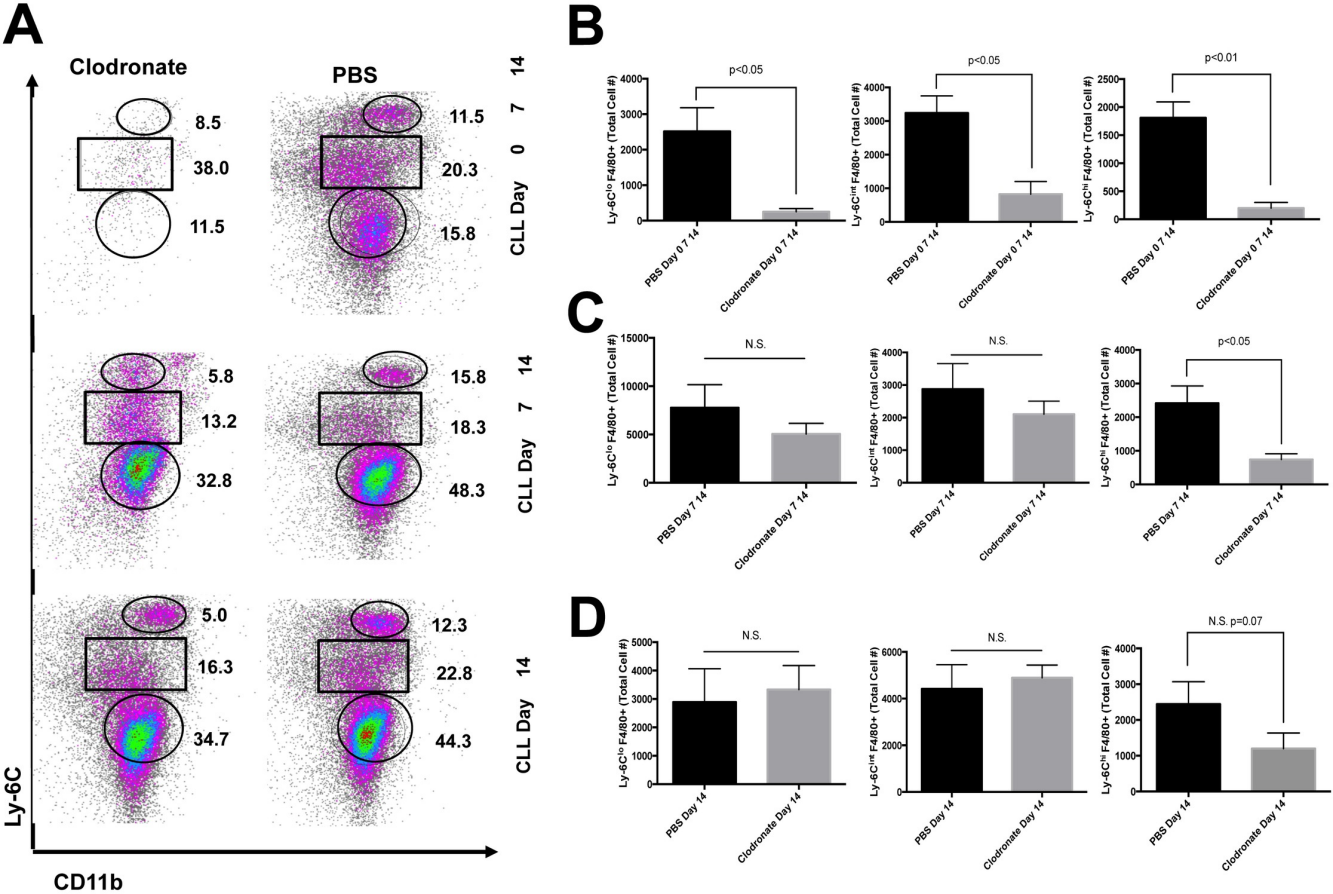
**Recruitment of Ly-6C+ monocytes is reduced with CLL treatment** (A) Clodronate-loaded liposome (CLL) treatment or PBS initiated at onset of MCD diet feeding leads to significant changes in Ly-6C+ cell populations. (B) A significant reduction in Ly-6C^hi^ (p<0.05), Ly-6C^int^ (p<0.05) and Ly-6C^lo^ (p<0.01) populations can be detected after 21 days of MCD diet feeding when CLL treatment is initiated at onset of MCD diet feeding. (C) Ly-6C^hi^ monocyte-derived macrophages are significantly reduced when CLL treatment is initiated at D7 of MCD diet feeding compared to PBS control (p<0.05). (D) No difference between cell populations is detected when CLL treatment is initiated 14 days after MCD diet feeding is started.

To understand the differences in leukocyte recruitment following CLL treatment we first assessed the infiltrating monocyte-derived macrophage populations (Fig 7A). Treating mice with CLL at the onset of MCD diet feeding resulted in a significant decrease in Ly-6C^lo^ (p<0.05), Ly-6C^int^ (p<0.05) and Ly-6C^hi^ (p<0.01) monocyte-derived macrophage populations compared to control mice (Fig 7B). When CLL treatment was initiated after 7 days of MCD diet feeding there was a significant decrease in Ly-6C^hi^ monocyte-derived macrophages (p<0.05) compared to control (Fig 7C). No differences in cell recruitment were detected when CLL was started after 14 days of MCD diet feeding (Fig 7D).

### Innate and adaptive lymphocytes are significantly reduced in the liver following CLL treatment at the initiation of MCD diet feeding

**Fig 8:**
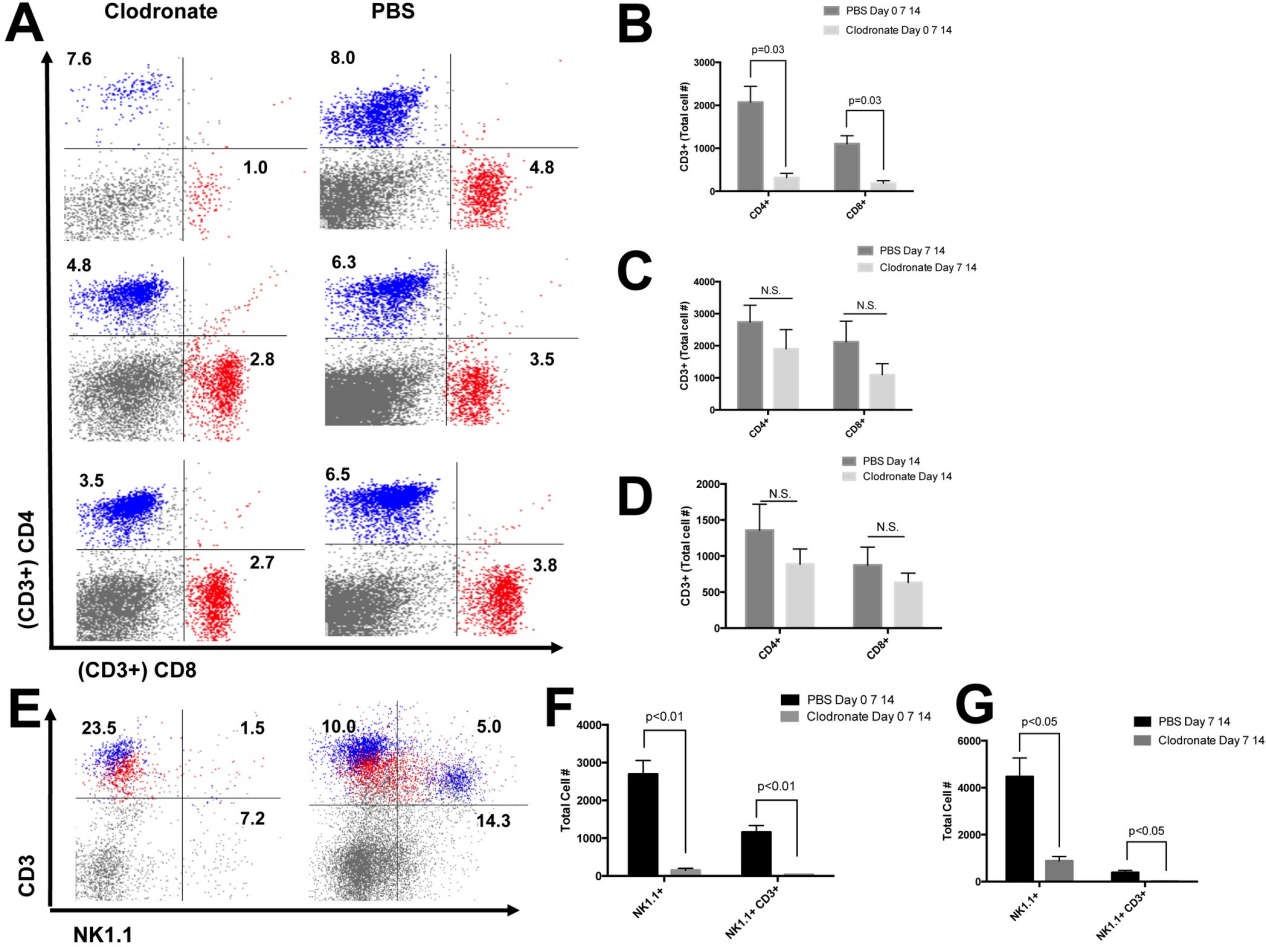
Reduction of leukocytes following CLL treatment. (A-B) Clodronate-loaded liposome (CLL) treatment or PBS initiated at onset of MCD diet feeding leads to significant changes in lymphoid cell populations (CD4+: blue CD8+: red). (C) A significant reduction in CD4+ (p<0.05) and CD8+ (p<0.05) T cell populations can be detected after 21 days of MCD diet feeding when CLL treatment is initiated at onset of MCD diet feeding. (D-E) No difference is detected in CD4+ and CD8+ lymphocytes when CLL is initiated at D7 or D14 following MCD diet treatment. (F-G) A significant reduction in NK cells (p<0.01) and NKT cells (p<0.01) can be detected after 21 days of MCD diet feeding when CLL treatment is initiated at onset of MCD diet feeding.

Although the recruitment of lymphoid cells appears to be a late event in MCD diet feeding we wanted to understand how depletion of macrophages at earlier time points would affect later recruitment of these cells (Fig 8A). Interestingly only mice treated with CLL at the onset of MCD diet feeding had significantly reduced numbers of CD4+ (p = 0.03) and CD8+ (p = 0.03) T cells compared to control mice (Fig 8B–8D). The presence of NK and iNKT cells is also reduced following CLL treatment (Fig 8E). The recruitment of NKT and iNKT cells was significantly reduced when CLL treatment was started at the onset of MCD diet feeding (Fig 8F, p<0.01). A similar pattern was observed when CLL treatment began after 7 days of MCD diet feeding (Fig 8G, p<0.05).

## Discussion

To understand the pathogenesis of NASH and develop effective treatment strategies, it is critical to study the role of Kupffer cells in initiating inflammation[4]. Although KCs have been established as a key cell type contributing to inflammation during NASH, studies are still required to define their role at the onset of NASH. Therefore our goal was to describe changes in KCs during the development of murine steatohepatitis and evaluate the response when KCs were depleted over the duration of NASH development in mice.

We observed a distinctive pattern of changes in both Kupffer cells and recruited monocytes during experimental steatohepatitis. Conventional KCs were lost early in the development of steatohepatitis and were subsequently replaced by a recruited monocyte-derived macrophage population. A similar phenomenon has been described in the case of bacterial infection with *Listeria monocytogenes* where KCs were found to undergo necroptosis and be replaced by bone marrow-derived monocytes responsible for tissue repair and re-establishment of homeostasis in the liver[26]. In a newly described model of diphtheria-toxin mediated KC depletion, bone marrow-derived monocytes filled the niche of liver-resident macrophages and adopted a transcriptional profile very similar to that of embryonically derived KCs in healthy mice[27]. We have recently shown that the loss of KCs during experimental NASH may be due in part to cell apoptosis mediated by gut-derived volatile organic compounds[28]. Understanding the changes in resident macrophages and recruited monocytes is key for both preventing inflammation and tissue damage as well as for the resolution of fibrosis. Depletion of Ly-6C^lo^ monocytes following chronic administration of carbon tetrachloride was shown to result in persistent fibrosis and delayed tissue-remodeling[11]. What remains to be examined is the physiological function(s) of recruited monocyte-derived macrophages replacing KCs during chronic inflammation as in the case of steatohepatitis.

Due to the changes in macrophage subsets we observed during MCD diet feeding we evaluated these populations at a transcriptomics level. Indeed, we found that the genes expressed in conventional KCs from healthy mice varied from that of Ly-6C^lo^ monocyte-derived macrophages isolated from mice with steatohepatitis even though they appeared to look similar when characterized by flow cytometry. We found decreased expression of transcription factors driving anti-inflammatory M2 polarization in Ly-6C^lo^ macrophages suggesting that the recruited cells may have a reduced immunoregulatory potential compared to resident Kupffer cells[29]. Recently it was found that the recruitment of GATA6^+^ resident peritoneal cavity macrophages to the liver was necessary to promote tissue repair following sterile burn injury[30]. This suggests that there is a critical difference between KCs in a healthy liver compared to macrophages that may be present in the tissue during inflammation and injury. The type of immune challenge has also been found to result in differences in function of proinflammatory monocytes recruited to the liver[8]. During persistent viral infection, recruited monocytes secreted numerous proinflammatory cytokines and chemokines whereas an injection of lipopolysaccharide resulted in highly phagocytic monocytes[8]. Careful analysis of these recruited populations during experimental steatohepatitis is required to fully understand their function.

Our analysis revealed that at the peak of liver injury we found the highest expression of CCR2 on monocyte-derived macrophages to co-express Ly-6C at a relatively low level. This is in contrast to some reports in the literature that have described ‘classical’ monocyte recruitment dependent on high levels of Ly-6C and CCR2 expression[7, 31]. In the case of chronic alcohol feeding in mice, Ly-6C^hi^ monocytes were found to adopt a Ly-6C^lo^ expression within the liver tissue suggesting that these cells retain a highly plastic phenotype and can modify their function in a given microenvironment[32]. Currently the phase 2b multinational clinical trial (CENTAUR) is underway to evaluate the anti-inflammatory and anti-fibrotic potential of a dual CCR2/CCR5 antagonist Cenicriviroc (CVC) in patients with NASH[10]. Continued study into the potential to modify the recruitment of proinflammatory cells and enhance tissue repair is required.

Following our microarray analysis we found that a number of genes influencing both cytokine-cytokine receptor interactions and chemokine-chemokine receptors interactions had changed during MCD diet feeding. We observed high levels of CX3CR1 expression on newly recruited monocyte-derived macrophages. This is in line with similar reports of TNF-α-producing CX3CR1+ dendritic cells recruited to the liver during experimental steatohepatitis[3]. Following KC depletion, we found a reduction in lymphocyte recruitment and reduced inflammation and liver injury likely mediated by the release of chemokines by macrophages in the liver. A role for CXCL16 release leading to the recruitment of CXCR6+ iNKT cells has been associated with progression of NASH in mice[33]. Recently expression of CXCR3 was found to be elevated in liver tissue from patients with NASH and on T lymphocytes in mice following a high fat-high cholesterol diet [34]. Importantly mice deficient in CXCR3 expression were protected against liver injury and inflammation thought to be due in part to a reduction in proinflammatory cytokine release from macrophages and reduced T cell accumulation [34]. Continued study of the modifications of key cytokines and chemokines is required to identify effective treatment strategies of NASH.

In our study depletion of KCs early on in MCD diet-induced steatohepatitis revealed a protective effect similar to previous reports[35]. In one case Kupffer cells were depleted prior to MCD diet feeding and five days after diet initiation and resulted in a reduction of hepatic TNF-α expression and decreased monocyte infiltration after ten days of dietary treatment[35]. This suggests a critical role for KCs in initiating an inflammatory response leading to NASH. This was further confirmed by our data where only KC depletion at the beginning of MCD diet treatment or after one week of diet protected the mice against NASH whereas KC depletion at day 14 did not change the degree of liver injury or inflammation. It is interesting to us that MCD diet feeding led to a reduction in KCs at early time points and at the same time depletion of tissue-resident macrophages with clodronate at the beginning of MCD diet feeding resulted in protection against steatohepatitis. This may provide evidence of cross-talk between different pools of tissue-resident macrophages leading to the orchestration of inflammation[27].

The etiology of NASH is still unclear and the development of an animal model that fully reflects the pathogenesis of NASH remains a challenge[16, 36]. While the MCD diet model induces a robust NASH phenotype including steatosis, inflammation and hepatocellular injury it is in the absence of other obesity traits[16]. Employing a ‘westernized’ diet containing high fat, high cholesterol and fructose can lead to weight gain, steatosis, ballooning hepatocytes and liver fibrosis similar to human NASH after six months of treatment[37]. Future studies utilizing ‘westernized’ diets consisting of high fat, cholesterol and/or simple carbohydrates such as sucrose or fructose should be conducted to evaluate changes in tissue-resident macrophages in murine models of obesity-induced fatty liver disease. The diets will need to be fed for extended periods though because our mice fed a HF/HS diet for 13 weeks did not show any indications of liver injury or change in KC number.

Although we did not observe a substantial change in inflammation when clodronate was administered following 14 days of MCD diet feeding this may be due to reports of KCs having reduced phagocytic function in chronic NAFLD [38]. Future studies are aimed at assessing a dose-dependent depletion of macrophages in the liver during active inflammation to evaluate the progression of disease.

Our examination of the development of experimental steatohepatitis also revealed changes in the recruitment of multiple immune cells including CD4+ and CD8+ T cells, NK and iNKT cells. Clodronate treatment resulted in the reduction of infiltrating CD8+ T cells likely contributing to further protection against hepatocellular injury as increased portal CD8+ T cells has been observed in patients with NASH[39]. At the peak of inflammation we observed a significant increase in iNKT cells within the liver. Release of osteopontin by macrophages has been associated with the recruitment of iNKT cells in mice with MCD diet-induced NASH and resultant fibrosis[40, 41]. The activation of hepatic stellate cells (HSCs) by the release of inflammatory molecules like osteopontin and transforming growth factor (TGF)-β1 from macrophages has also been linked to the promotion of liver fibrosis[42, 43].

In conclusion we have identified critical changes in resident and recruited macrophages in the liver during experimental steatohepatitis. Based on gene expression analysis it would suggest that there are numerous functions for macrophages in chronic liver disease and their regulation is a dynamic process. Care should be taken when characterizing these cell types in the liver.

## Supporting Information

S1 Fig Fibrosis development following six weeks of MCD diet treatment. (A) There was a significant increase in α-SMA expression in whole liver tissue from mice fed MCD diet for six weeks compared to three weeks measured by real-time qPCR (p = 0.05). (B) Picrosirius red staining of a representative liver section from a mouse fed MCD diet for three weeks demonstrates steatosis and inflammation but very little fibrosis development. (C) Following six weeks of MCD diet treatment mice begin to develop fibrosis in a ‘chicken wire’ pattern as observed with Picrosirius red staining of a representative liver section. 20x magnification, PT: portal tract, CV: central vein.
(TIF)

S2 Fig Development of NAFLD in mice following HF/HS diet treatment. (A) Following 13 weeks of HF/HS diet feeding, there was no difference in the number of Ly-6C+F4/80+ macrophages in the liver compared to standard chow fed mice. In contrast, three weeks of MCD diet treatment lead to a reduction in tissue-resident macrophages and a substantial recruitment of F4/80+ CCR2+ cells. (B) Representative liver section from a mouse fed standard chow diet. (C) Representative liver section from a mouse fed HF/HS diet for 13 weeks. While the mice develop steatosis it is in the absence of inflammation. (D) Representative liver section from a mouse fed MCD diet for three weeks. In addition to steatosis there is evidence of ballooning hepatocytes and infiltrating leukocytes. (E) There was a significant reduction in F4/80+ tissue-resident macrophages (*** p<0.001) and a significant increase in F4/80+CCR2+ recruited macrophages (* p<0.05) in mice fed MCD diet for three weeks as measured by flow cytometry. (F) There was a significant increase in serum ALT in mice fed MCD diet (**** p<0.0001) for three weeks compared to standard chow and HF/HS diet treatments whereas there was no difference in serum ALT levels between mice fed HF/HS diet and standard chow. 20x magnification, PT: portal tract, CV: central vein.
(TIF)
