# Supplementary figures and images for: Kupffer Cells Undergo Fundamental Changes during the Development of Experimental NASH and Are Critical in Initiating Liver Damage and Inflammation
Source: PLoS One. 2016 Jul 25;11(7):e0159524. doi: 10.1371/journal.pone.0159524 (PMC4959686; doi:10.1371/journal.pone.0159524)

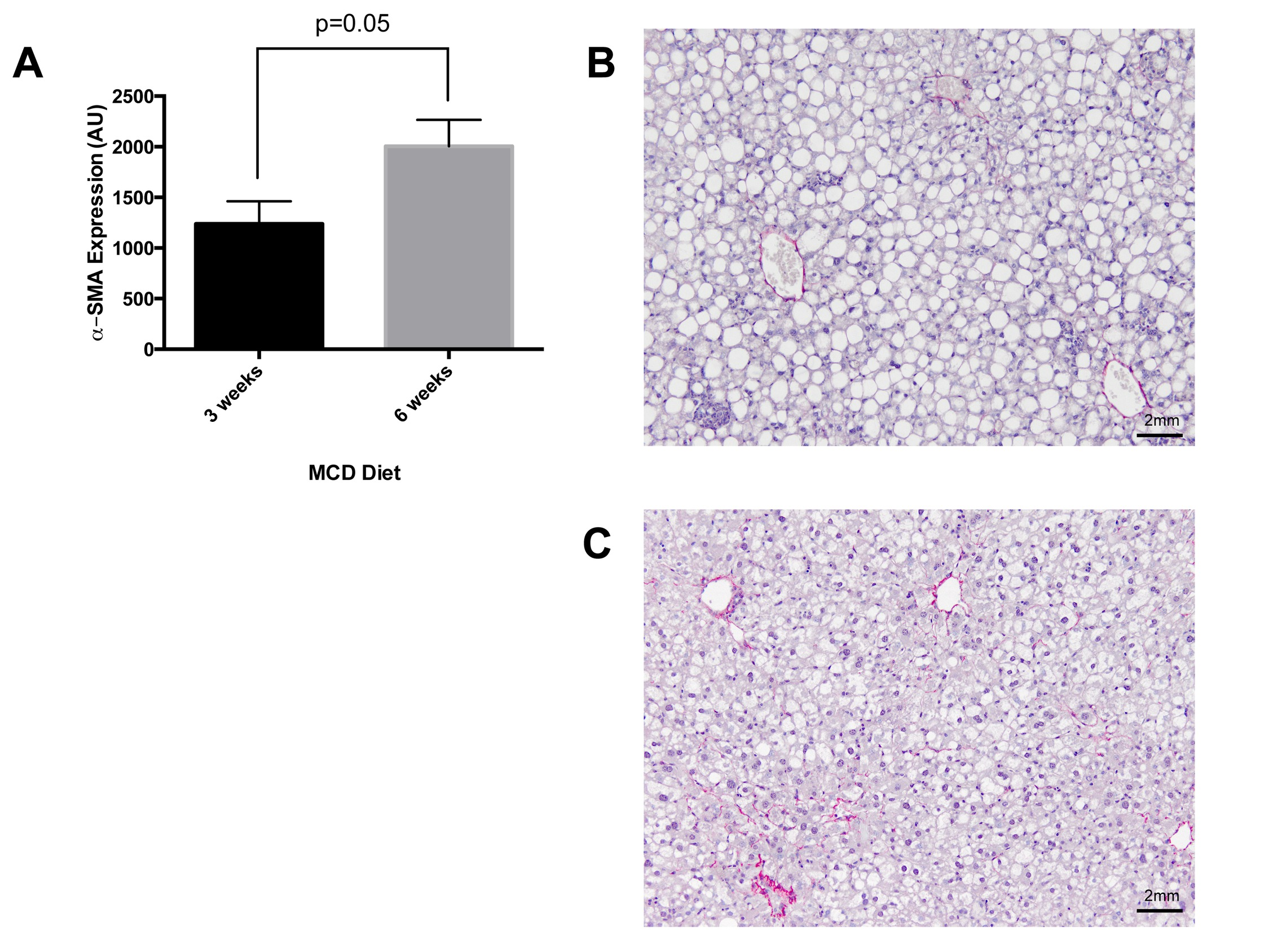

Supplement: S1 Fig — (A) There was a significant increase in α-SMA expression in whole liver tissue from mice fed MCD diet for six weeks compared to three weeks measured by real-time qPCR (p = 0.05). (B) Picrosirius red staining of a representative liver section from a mouse fed MCD diet for three weeks demonstrates steatosis and inflammation but very little fibrosis development. (C) Following six weeks of MCD diet treatment mice begin to develop fibrosis in a ‘chicken wire’ pattern as observed with Picrosirius red staining of a representative liver section. 20x magnification, PT: portal tract, CV: central vein. (TIF) [file pone.0159524.s001.tif]

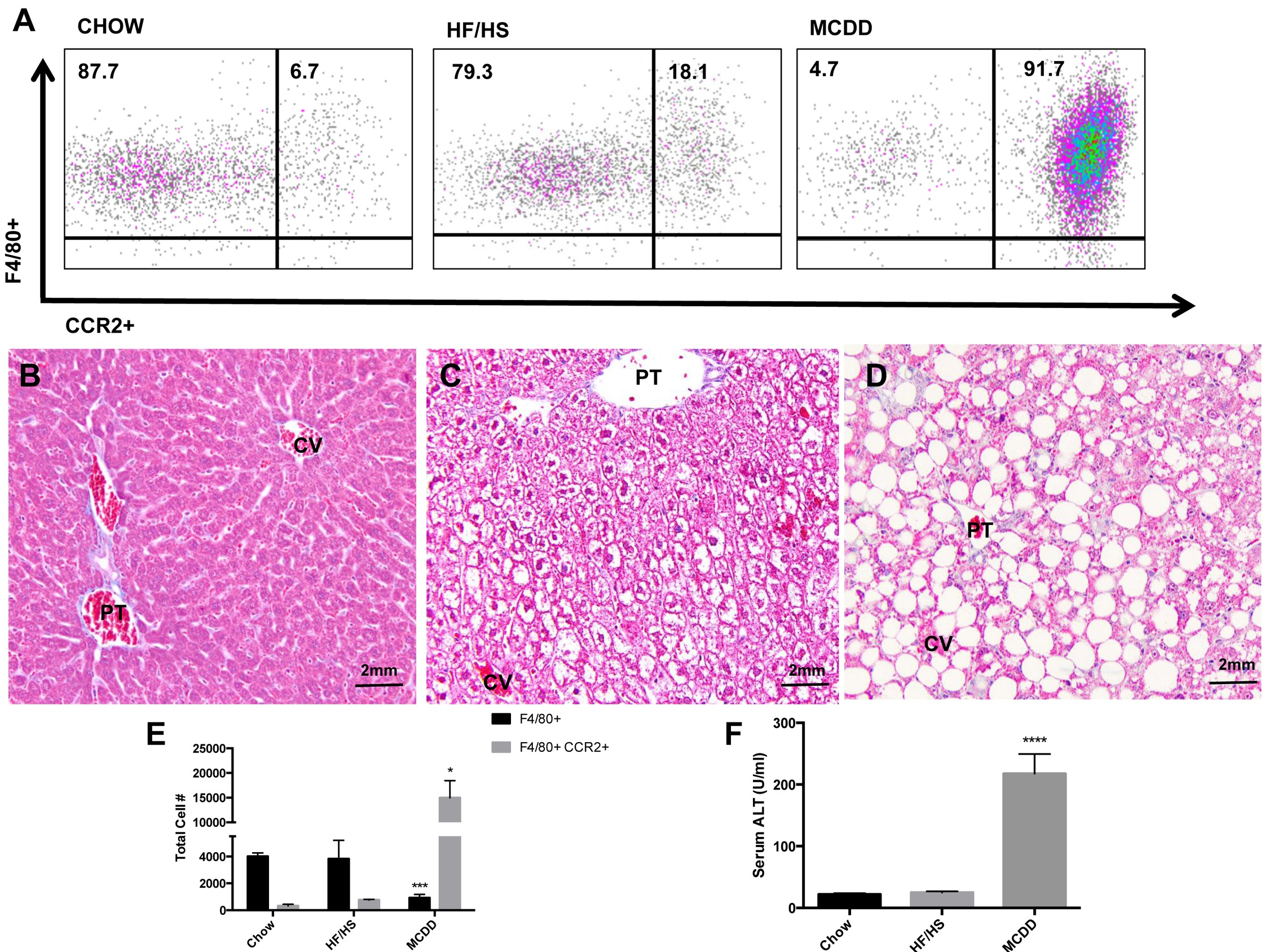

Supplement: S2 Fig — (A) Following 13 weeks of HF/HS diet feeding, there was no difference in the number of Ly-6C+F4/80+ macrophages in the liver compared to standard chow fed mice. In contrast, three weeks of MCD diet treatment lead to a reduction in tissue-resident macrophages and a substantial recruitment of F4/80+ CCR2+ cells. (B) Representative liver section from a mouse fed standard chow diet. (C) Representative liver section from a mouse fed HF/HS diet for 13 weeks. While the mice develop steatosis it is in the absence of inflammation. (D) Representative liver section from a mouse fed MCD diet for three weeks. In addition to steatosis there is evidence of ballooning hepatocytes and infiltrating leukocytes. (E) There was a significant reduction in F4/80+ tissue-resident macrophages (*** p<0.001) and a significant increase in F4/80+CCR2+ recruited macrophages (* p<0.05) in mice fed MCD diet for three weeks as measured by flow cytometry. (F) There was a significant increase in serum ALT in mice fed MCD diet (**** p<0.0001) for three weeks compared to standard chow and HF/HS diet treatments whereas there was no difference in serum ALT levels between mice fed HF/HS diet and standard chow. 20x magnification, PT: portal tract, CV: central vein. (TIF) [file pone.0159524.s002.tif]
